# The origin of the prion agent of kuru: molecular and biological strain typing

**DOI:** 10.1098/rstb.2008.0069

**Published:** 2008-11-27

**Authors:** Jonathan D.F. Wadsworth, Susan Joiner, Jacqueline M. Linehan, Emmanuel A. Asante, Sebastian Brandner, John Collinge

**Affiliations:** MRC Prion Unit and Department of Neurodegenerative Disease, UCL Institute of Neurology, National Hospital for Neurology and NeurosurgeryQueen Square, London WC1N 3BG, UK

**Keywords:** kuru, prion, spongiform encephalopathy

## Abstract

Kuru is an acquired human prion disease that primarily affected the Fore linguistic group of the Eastern Highlands of Papua New Guinea. The central clinical feature of kuru is progressive cerebellar ataxia and, in sharp contrast to most cases of sporadic Creutzfeldt–Jakob disease (CJD), dementia is a less prominent and usually late clinical feature. In this regard, kuru is more similar to variant CJD, which also has similar prodromal symptoms of sensory disturbance and joint pains in the legs and psychiatric and behavioural changes. Since a significant part of the clinicopathological diversity seen in human prion disease is likely to relate to the propagation of distinct human prion strains, we have compared the transmission properties of kuru prions with those isolated from patients with sporadic, iatrogenic and variant CJD in both transgenic and wild-type mice. These data have established that kuru prions have prion strain properties equivalent to those of classical (sporadic and iatrogenic) CJD prions but distinct from variant CJD prions. Here, we review these findings and discuss how peripheral routes of infection and other factors may be critical modifiers of the kuru phenotype.

## 1. Introduction

Human prion diseases are fatal neurodegenerative disorders that include Creutzfeldt–Jakob disease (CJD), Gerstmann–Sträussler–Scheinker disease, fatal familial insomnia, kuru and variant CJD (vCJD; Collinge 2001, 2005; Wadsworth & Collinge 2007). Their central feature is the post-translational conversion of host-encoded, cellular prion protein (PrP^C^) to an abnormal isoform, designated PrP^Sc^ (Prusiner 1982; Collinge 2001). Substantial evidence indicates that an abnormal PrP isoform is the principal, if not the sole, component of the transmissible infectious agent, or prion (Prusiner 1982; Collinge 2001; Weissmann 2004; Collinge & Clarke 2007). Human prion diseases are biologically unique in that the disease process can be triggered through inherited germ line mutations in the human prion protein gene (*PRNP*), infection (by inoculation, or in some cases by dietary exposure) with prion-infected tissue or by rare sporadic events that generate PrP^Sc^ (Collinge 2001, 2005; Wadsworth *et al.* 2003; Wadsworth & Collinge 2007).

Human prion diseases are associated with a range of clinical presentations and are classified by both clinicopathological syndrome and aetiology with sub-classification according to molecular criteria (Collinge 1997, 2005; Collinge & Palmer 1997; Wadsworth *et al.* 2003). Approximately 85 per cent of cases occur sporadically as CJD (sporadic CJD) at a rate of 1–2 cases per million population per year across the world, with an equal incidence in men and women (Brown *et al.* 1987; Collinge 2001, 2005; Wadsworth *et al.* 2003; Collins *et al.* 2006). Approximately 15 per cent of human prion disease is associated with autosomal dominant pathogenic *PRNP* mutations and to date over 30 mutations have been described (Collinge 2001, 2005; Kovacs *et al.* 2002; Wadsworth *et al.* 2003; Mead 2006). Iatrogenic forms of prion disease have occurred most frequently due to the transmission of CJD prions via contaminated growth hormone derived from human cadavers, or by implantation of contaminated dura mater grafts (Brown *et al.* 1992, 2000). Iatrogenic prion disease has also resulted from transmission of CJD prions during corneal transplantation, contaminated electroencephalographic (EEG) electrode implantation and surgical operations using contaminated instruments or apparatus (Brown *et al.* 1992, 2000). Acquired prion disease in humans due to a dietary origin has resulted in kuru, an epidemic prion disease principally of the Fore linguistic group of the Eastern Highlands of Papua New Guinea, which was transmitted during mortuary feasts when deceased relatives were consumed by close relatives as a mark of respect and mourning (Alpers 1987; Collinge & Palmer 1997; Mead *et al.* 2003), and vCJD in the United Kingdom and other countries caused by human exposure to BSE prions from cattle (Collinge *et al.* 1996; Bruce *et al.* 1997; Hill *et al.* 1997; Collinge 1999; Asante *et al.* 2002). Kuru demonstrates that incubation periods of infection with human prions can exceed 50 years (Collinge *et al.* 2006) and these data indicate that the parameters of any vCJD epidemic cannot yet be predicted with confidence (Collinge 1999; Frosh *et al.* 2004; Hilton *et al.* 2004; Collinge *et al.* 2006).

Human PrP has a common polymorphism at residue 129 (encoding either methionine or valine) and homozygosity confers genetic susceptibility to both sporadic and acquired forms of CJD (Collinge *et al.* 1991; Palmer *et al.* 1991; Windl *et al.* 1996; Lee *et al.* 2001; Mead *et al.* 2003; Collins *et al.* 2006). The effect of codon 129 is most strikingly observed in vCJD where all clinical cases studied so far have been methionine homozygotes (Collinge 2005; Wadsworth & Collinge 2007). Codon 129 genotype also shows a pronounced effect on kuru incubation periods and susceptibility (Cervenáková *et al.* 1999; Lee *et al.* 2001; Mead *et al.* 2003; Collinge *et al.* 2006) and the clear survival advantage for *PRNP* codon 129 heterozygotes provides a powerful basis for balancing selection in the Fore where the majority of kuru-exposed survivors are *PRNP* 129 heterozygotes (Mead *et al.* 2003).

The remarkable degree of clinical and neuropathological heterogeneity observed across the spectrum of human prion disease has yet to be fully explained. However, it has been clear for many years that distinct isolates, or strains, of prions can be propagated in the same host and these are biologically recognized by distinctive clinical and pathological features in inoculated laboratory animals (Collinge 2001; Hill & Collinge 2001; Bruce 2003; Collinge & Clarke 2007). It is therefore probable that a significant proportion of clinicopathological heterogeneity seen in human prion diseases relates to the propagation of distinct human prion strains (Collinge 2001; Collinge & Clarke 2007). Genetic factors also influence prion strain selection through the coding sequence of the host prion protein gene (Collinge 1999, 2001; Hill & Collinge 2003; Wadsworth *et al.* 2004; Collinge & Clarke 2007) and other unknown non-*PRNP* genetic factors have also been revealed (Stephenson *et al.* 2000; Lloyd *et al.* 2001, 2004*a*; Asante *et al.* 2002).

The origins of kuru have remained somewhat obscure; however, in the absence of a pathogenic *PRNP* mutation associated with kuru (Lee *et al.* 2001; Mead *et al.* 2003), or any evidence for an animal origin, the most plausible hypothesis for the source of kuru is through chance consumption of an individual with sporadic CJD (Alpers & Rail 1971). However, the clinical features of kuru are remarkably stereotyped and distinct from the majority of cases of sporadic CJD. A prodrome and three clinical stages consisting of an ambulatory stage, a sedentary stage and a tertiary stage have been described in which progressive cerebellar ataxia is the dominant clinical feature, with dementia being a late and less prominent characteristic (Alpers 1987; Brown *et al.* 1994; Collinge & Palmer 1997; Collinge 2005; Collinge *et al.* 2006). In this regard, kuru is more reminiscent of variant CJD in which early symptoms of peripheral sensory disturbance, joint pains, psychiatric and behavioural changes are seen prior to overt dementia (Collinge 2005; Collinge *et al.* 2006). Because clinicopathological diversity in human prion diseases may relate to the propagation of different prion strains, we have compared the transmission properties of kuru prions with sporadic, iatrogenic and variant CJD prions in transgenic and wild-type mice. These data show that kuru prions are distinct from vCJD prions and have molecular characteristics and transmission properties closely similar to classical CJD prions (Wadsworth *et al.* 2008). Here, we review these findings and discuss their implications for understanding the clinicopathological phenotype of kuru.

## 2. Molecular strain typing studies

Within the framework of the protein-only hypothesis of prion propagation, the different phenotypes associated with prion strains are thought to be determined by the propagation of distinct PrP^Sc^ isoforms with divergent physicochemical properties (Bessen & Marsh 1994; Collinge *et al.* 1996; Telling *et al.* 1996; Prusiner 1998; Safar *et al.* 1998; Collinge 2001; Hill & Collinge 2001; Gambetti *et al.* 2003; Collinge & Clarke 2007). To date, we have identified four major types of human PrP^Sc^ associated with sporadic and acquired human prion diseases that can be differentiated on immunoblots after limited proteinase K digestion of brain homogenates (Collinge *et al.* 1996; Wadsworth *et al.* 1999; Hill *et al.* 2003). The different PrP fragment sizes seen on immunoblots following treatment with proteinase K (corresponding to amino-terminally truncated cleavage products generated from di-, mono- or non-glycosylated PrP^Sc^) suggest that there are several different human PrP^Sc^ conformations, referred to as molecular strain types and these can be further classified by the ratio of the three PrP fragments seen after protease digestion. PrP^Sc^ types 1–3 are seen in classical (sporadic or iatrogenic) CJD brain, while type 4 PrP^Sc^ is uniquely seen in vCJD brain (Collinge *et al.* 1996; Wadsworth *et al.* 1999; Hill *et al.* 2003). Polymorphism at human PrP residue 129 places constraints upon the propagation of distinct human PrP^Sc^ types and these data provide a molecular basis for this polymorphism acting as a major factor influencing both prion disease susceptibility and phenotype in humans (Collinge 2001, 2005; Mead *et al.* 2003; Wadsworth & Collinge 2007). To date, human PrP^Sc^ types 1 and 4 have been found only in humans homozygous for *PRNP* codon 129 methionine; type 3 PrP^Sc^ is seen almost exclusively in individuals with at least one valine allele, while type 2 PrP^Sc^ has been commonly observed in all codon 129 genotypes (Collinge *et al.* 1996; Wadsworth *et al.* 1999; Hill *et al.* 2003). A simpler molecular classification of classical CJD is also in use, based on subdivision into only two molecular sub-types by fragment size (Parchi *et al.* 1996, 1999). International agreement on systematic classification of molecular strain types in human prion disease has not yet been achieved (Wadsworth & Collinge 2007).

**Figure 1:**
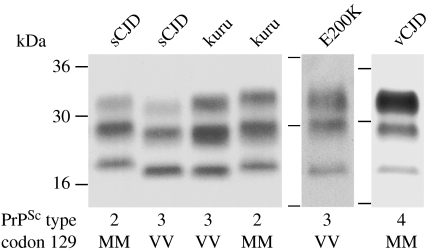
PrP^Sc^ strain types in human prion disease. Immunoblots of proteinase K digested brain homogenates from patients with human prion disease. Disease aetiology of each brain sample is designated above each lane and the PrP^Sc^ type (using the London classification of human PrP^Sc^ types; Collinge *et al.* 1996; Hill *et al.* 2003, 2006) and *PRNP* codon 129 genotype of the patient (M, methionine; V, valine) are designated below. Immunoblots were developed with anti-PrP monoclonal antibody 3F4 using a chemiluminescent substrate.

Recently, we have reported investigation of frontal cortex brain samples from three neuropathologically confirmed patients with kuru (Wadsworth *et al.* 2008). These isolates were provided by the late Dr Clarence J Gibbs of the National Institutes of Health, Bethesda MD, USA, and belonged to a cohort of 18 kuru patient brain samples that were successfully transmitted to non-human primates at the National Institutes of Health between 1963 and 1993 (Brown *et al.* 1994). All patients in this series were reported to have the usual stereotyped clinical progression of kuru with progressive ataxia in the absence of early cognitive impairment (Brown *et al.* 1994). Our analysis of these isolates showed protease-resistant PrP fragment patterns corresponding to either the type 2 or 3 PrP^Sc^ pattern that we have observed in sporadic and iatrogenic CJD (Wadsworth *et al.* 2008). Two patients propagated type 3 PrP^Sc^ and were homozygous for valine at *PRNP* codon 129; the other patient propagated type 2 PrP^Sc^ and was homozygous for methionine at *PRNP* codon 129 (Wadsworth *et al.* 2008; figure 1). The glycoform ratios of protease-resistant PrP fragments in these kuru isolates were all similar to the PrP glycoform ratios seen in classical CJD rather than the distinctive PrP glycoform ratios seen in either vCJD or inherited prion disease caused by *PRNP* point mutations (Hill *et al.* 2003, 2006; Wadsworth *et al.* 2006; Wadsworth & Collinge 2007; figure 1). Collectively, these data are consistent with the findings of Gambetti and colleagues who have also reported close similarity of the PrP^Sc^ types propagated in kuru and classical CJD (Parchi *et al.* 1997, 2000).

## 3. Transmission rates of human prions in transgenic and wild-type mice

Over the last 15 years, we have investigated the transmission properties of prions from a cohort of patients with neuropathologically confirmed sporadic, iatrogenic and variant CJD in transgenic mice expressing only human PrP with either valine (V) or methionine (M) at residue 129 and in wild-type mice (Collinge *et al.* 1996; Hill *et al.* 1997; Asante *et al.* 2002, 2006; Wadsworth *et al.* 2004, 2008).

**Figure 2:**
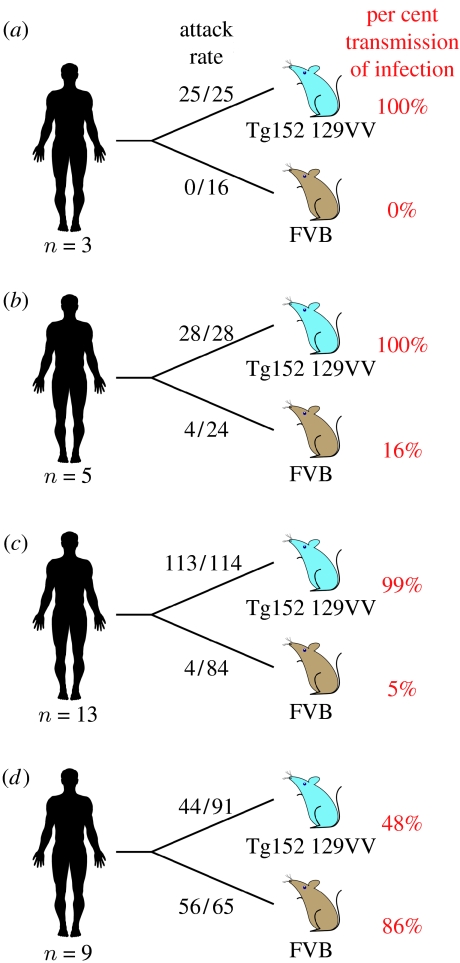
Summary of rates of transmission of human prions to transgenic and wild-type mice. Brain homogenate from patients with neuropathologically confirmed (*a*) kuru, (*b*) iatrogenic CJD, (*c*) sporadic CJD and (*d*) variant CJD were inoculated intra-cerebrally into transgenic mice homozygous for a human PrP 129V transgene array and murine PrP null alleles (*Prnp*^o/o^) designated Tg(HuPrP129V^+/+^ *Prnp*^o/o^)-152 mice (129VV Tg152 mice; Collinge *et al.* 1995), and into wild-type FVB/NHsd mice (genotype *Prnp*^a^). The number of prion disease isolates examined is designated below each schematic patient. Attack rate is defined as the total number of both clinically affected and sub-clinically infected mice as a proportion of the number of inoculated mice. In mice that were asymptomatic, sub-clinical prion infection was assessed by immunoblotting and/or immunohistochemical examination of brain. Primary data are described in references (Collinge *et al.* 1995, 1996; Hill *et al.* 1997; Wadsworth *et al.* 2004, 2008).

**Figure 3:**
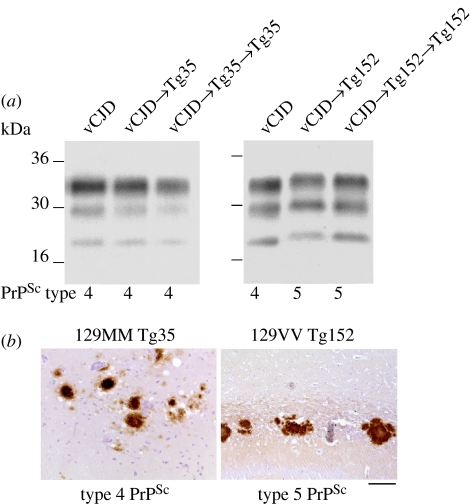
Summary of transmission of vCJD prions to transgenic mice expressing human PrP. Primary and secondary transmission of vCJD prions in transgenic Tg(HuPrP129M^+/+^ *Prnp*^o/o^)-35 mice (Tg35) results in faithful propagation of type 4 PrP^Sc^ and the occurrence of abundant florid PrP plaques throughout the cortex which are the neuropathological hallmark of vCJD. By contrast, primary and secondary transmission of vCJD prions to transgenic Tg(HuPrP129V^+/+^ *Prnp*^o/o^)-152 mice (Tg152) results in the propagation of a novel prion strain characterized by type 5 PrP^Sc^ and the occurrence of large, non-florid PrP plaques in the corpus callosum. (*a*) Representative immunoblots of proteinase K-treated brain homogenates from variant CJD and transgenic mice analysed with anti-PrP monoclonal antibody 3F4. The identity of the brain sample is designated above each lane with the type of PrP^Sc^ present in the sample designated below (using the London classification of human PrP^Sc^ types; Collinge *et al.* 1996; Hill *et al.* 2003; Wadsworth *et al.* 2004). (*b*) Representative immunohistochemical analysis of transgenic mouse brain showing abnormal PrP-positive plaques stained with anti-PrP monoclonal antibody 3F4. Scale bar, 100 μm.

Transgenic mice expressing human PrP 129 valine, but not mouse PrP (129VV Tg152 mice), lack a transmission barrier to classical (sporadic and iatrogenic) CJD prions, regardless of the codon 129 genotype of the inoculum (Collinge *et al.* 1995, 1996; Hill *et al.* 1997; Wadsworth *et al.* 2008; figure 2). These transmissions are characterized by approximately 100 per cent attack rates of prion infection producing clinical prion disease with similar short incubation periods of approximately 200 days (Collinge *et al.* 1995, 1996; Hill *et al.* 1997; Wadsworth *et al.* 2008; figure 2). By contrast, primary challenge of 129VV Tg152 mice with vCJD prions is characterized by a substantial transmission barrier with only approximately 50 per cent of inoculated mice becoming infected (Hill *et al.* 1997; Wadsworth *et al.* 2004, 2008; figure 2). Affected vCJD-inoculated Tg152 mice propagate a novel prion strain associated with type 5 PrP^Sc^ (Hill *et al.* 1997; Wadsworth *et al.* 2004, 2008; figure 3) which fails to adapt after secondary passage in further 129VV Tg152 mice (Wadsworth *et al.* 2004). In wild-type FVB/NHsd mice (genotype *Prnp*^a^; Lloyd *et al.* 2004*b*), the relative transmission efficiencies of classical and variant CJD prions are quite different (Collinge *et al.* 1995, 1996; Hill *et al.* 1997; Wadsworth *et al.* 2004, 2008). Classical CJD prions transmit infection to FVB/NHsd mice only occasionally, at long and variable incubation periods, whereas vCJD prions transmit infection far more efficiently, although incubation periods are prolonged and variable (Hill *et al.* 1997; Asante *et al.* 2002; Wadsworth *et al.* 2004, 2008; figure 2).

Recently, we reported that kuru prions have transmission rates in 129VV Tg152 and FVB/NHsd mice closely similar to those of classical CJD prions rather than those of vCJD prions (Wadsworth *et al.* 2008; figure 2). In 129VV Tg152 mice, kuru isolates produce 100 per cent attack rates of prion infection causing clinical prion disease with closely similar mean incubation periods of approximately 200 days (Wadsworth *et al.* 2008; figure 2). In sharp contrast, no evidence for prion infection is seen in kuru-inoculated FVB/NHsd mice after prolonged observation periods (Wadsworth *et al.* 2008; figure 2). These data are in agreement with those of Brown and colleagues who showed that experimental transmission rates of kuru prions in non-human primates were also closely similar to classical CJD isolates and distinct from inherited forms of prion disease (Brown *et al.* 1994).

## 4. Molecular and neuropathological phenotypes in transgenic mice propagating human prions

The hypothesis that alternative conformations or assembly states of PrP provide the molecular substrate for clinicopathological heterogeneity seen in human prion diseases (and that this relates to the existence of distinct human prion strains) has been strongly supported by molecular and neuropathological analysis of human prion transmissions to conventional and transgenic mice. Transgenic mice expressing only human PrP have shown that residue 129 polymorphism constrains both the propagation of distinct human PrP^Sc^ conformers and the occurrence of associated patterns of neuropathology (Collinge *et al.* 1996; Hill *et al.* 1997; Asante *et al.* 2002, 2006; Wadsworth *et al.* 2004, 2008; figure 3). Human PrP^Sc^ types 1 and 4 that are seen only in *PRNP* codon 129 methionine homozygous patients do not replicate faithfully in 129VV Tg152 mice. Type 1 PrP^Sc^ converts to a PrP^Sc^ type with maintained glycoform ratio but a type 2 fragment size (Collinge *et al.* 1996) and type 4 PrP^Sc^ converts to type 5 PrP^Sc^ with the same glycoform ratio as type 4 PrP^Sc^ but a type 2 PrP^Sc^-like fragment size (Hill *et al.* 1997; Wadsworth *et al.* 2004; figure 3). By contrast, in isolates examined to date, PrP^Sc^ types 2 and 3 appear to propagate faithfully in 129VV Tg152 mice with no apparent change in proteolytic fragment size (Collinge *et al.* 1996; Wadsworth *et al.* 2008). These data are consistent with a conformational selection model of prion transmission barriers (Collinge 1999, 2001; Hill & Collinge 2003; Wadsworth *et al.* 2004; Collinge & Clarke 2007) and strongly support the ‘protein only’ hypothesis of prion infectivity by suggesting that prion strain variation is encoded by a combination of PrP conformation and glycosylation (Collinge 2001; Collinge & Clarke 2007).

**Figure 4:**
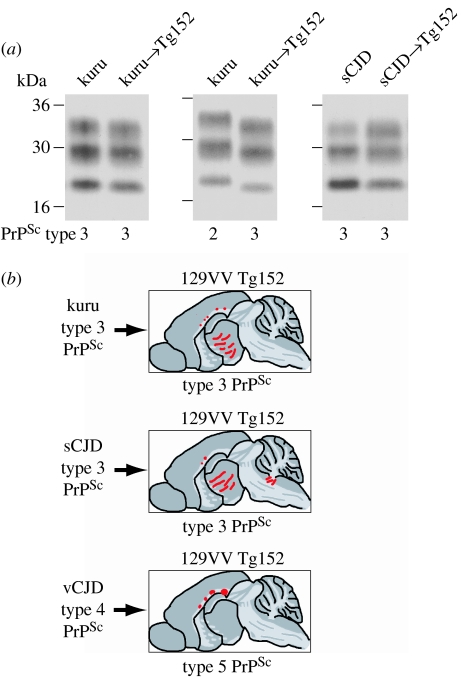
Summary of molecular and neuropathological phenotypes in transgenic mice. (*a*) Molecular strain typing of human prion transmissions to mice. Immunoblots of proteinase K-digested brain homogenates from human patients or transgenic mice analysed by enhanced chemiluminescence with anti-PrP monoclonal antibody 3F4. The provenance of each brain sample is designated above each lane and the type of human PrP^Sc^ detected in each sample (using the London classification of human PrP^Sc^ types; Collinge *et al.* 1996; Hill *et al.* 2003) is designated below. (*b*) Neuropathological analysis of transgenic mouse brain. An equivalent pattern of neuropathology is seen in 129VV Tg152 mice that propagate type 3 PrP^Sc^ following primary transmission of kuru prions or sporadic CJD prions, which is distinct from 129VV Tg152 mice that propagate type 5 PrP^Sc^ following primary transmission of vCJD prions. Sketches represent the regional distribution of abnormal PrP deposition in transgenic mouse brain visualized with anti-PrP monoclonal antibody ICSM 35; bars, diffuse synaptic PrP deposition and circles, PrP plaques.

In agreement with the close similarities of both PrP^Sc^ type and transmission rates of kuru prions and classical CJD prions, type 3 PrP^Sc^ from kuru or sporadic CJD brain propagated faithfully in 129VV Tg152 mice (Wadsworth *et al.* 2008; figure 4). In these mice, abnormal PrP deposition is characterized by a predominantly diffuse pattern of PrP staining throughout the thalamus and hypothalamus, occasionally accompanied by small PrP plaques in the corpus callosum (Wadsworth *et al.* 2008). Interestingly, the same pattern of neuropathology and the propagation of type 3 PrP^Sc^ was also observed in 129VV Tg152 mice inoculated with the type 2 PrP^Sc^ kuru isolate (Wadsworth *et al.* 2008; figure 4). This switch in molecular strain type may reflect the behaviour of sub-types of sporadic CJD-like prions associated with type 2 PrP^Sc^ and codon 129 methionine when crossing a *PRNP* codon 129 transmission barrier; however, further transmissions of CJD isolates with this molecular strain type will be required to investigate this directly (Wadsworth *et al.* 2008).

The pattern of neuropathology associated with the propagation of type 3 PrP^Sc^ in 129VV Tg152 mice contrasts sharply with that seen in vCJD-inoculated 129VV Tg152 mice propagating type 5 PrP^Sc^. In both clinically affected and sub-clinically infected 129VV Tg152 mice, the propagation of type 5 PrP^Sc^ is only accompanied by the occurrence of large, non-florid, PrP plaques in the corpus callosum with an absence of PrP plaques or diffuse PrP deposition in other brain areas (Wadsworth *et al.* 2004, 2008; figure 4). Thus, kuru- and sporadic CJD-inoculated 129VV Tg152 mice show a closely similar pattern of neuropathology that is distinct from the pattern seen in vCJD-inoculated mice.

## 5. Determinants of kuru phenotype

Molecular and biological strain typing studies have established that kuru prions have molecular strain types (Parchi *et al.* 1997, 2000; Wadsworth *et al.* 2008) and transmission properties (Brown *et al.* 1994; Wadsworth *et al.* 2008) equivalent to those of classical CJD prions rather than vCJD prions or inherited forms of prion disease. These data strongly support the hypothesis that kuru originated from chance consumption of an individual with sporadic CJD (Alpers & Rail 1971).

Despite the close molecular and biological similarity of kuru prions and sporadic CJD prions, both the clinical presentation and the neuropathology of kuru are distinct from the majority of patients with sporadic CJD. While rapidly progressive dementia is the defining clinical feature of approximately 70 per cent of cases of sporadic CJD (Brown *et al.* 1987; Parchi *et al.* 1996, 1999; Collinge 2001, 2005; Hill *et al.* 2003; Wadsworth *et al.* 2003; Collins *et al.* 2006), the central clinical feature of kuru is progressive cerebellar ataxia with dementia appearing as a late and less prominent feature (Alpers 1987; Brown *et al.* 1994; Collinge & Palmer 1997; Collinge 2005; Collinge *et al.* 2006). Furthermore, although the neuropathological changes seen in kuru lie within the spectrum of those seen in sporadic CJD, unicentric PrP plaques are unusually prominent and widespread (Hainfellner *et al.* 1997; McLean *et al.* 1998). Because a progressive cerebellar syndrome and the occurrence of kuru-type plaques reminiscent of kuru are also notable features of iatrogenic CJD resulting from peripheral exposure to sporadic CJD prions (Brown *et al.* 1992, 2000, 2006; Billette de Villemeur *et al.* 1994; Will 2003), these observations suggest that cerebellar onset and subsequent neuropathological changes in kuru may be significantly determined by peripheral routes of infection (predominantly dietary), rather than prion strain type. In this context, although the clinical presentation of most sporadic CJD patients is distinct from kuru, atypical forms of sporadic CJD are well recognized (Gomori *et al.* 1973; Brown *et al.* 1984; Parchi *et al.* 1996, 1999; Hill *et al.* 2003; Collins *et al.* 2006), and notably a relatively rare sub-type of sporadic CJD associated with long clinical duration and progressive ataxia also shows prominent kuru-type PrP plaques (Parchi *et al.* 1996, 1999; Hill *et al.* 2003). As the aetiology of sporadic CJD suggests involvement of a stochastic process such as somatic *PRNP* mutation (Brown *et al.* 1987; Collinge 2001; Wadsworth *et al.* 2006; Mead *et al.* 2007), it seems probable that part of the phenotypic and neuropathological heterogeneity seen in sporadic CJD may be related to peripheral versus central initiation of prion replication. Clearly, this might also be applicable in inherited forms of prion disease where extensive phenotypic variability is observed (Kovacs *et al.* 2002; Collinge 2005; Mead 2006; Wadsworth & Collinge 2007).

In addition to the route of exposure, other factors may also influence the neuropathology and clinical features of kuru. Age is an important determinant of survival in sporadic and inherited prion diseases (Pocchiari *et al.* 2004; Mead *et al.* 2006) and the marked difference in mean age of kuru and sporadic CJD patients may account for some of the differences that distinguish kuru from the majority of sporadic CJD cases. Genetic modifiers of prion disease may also be important. A number of non-*PRNP* genetic loci that exert a major influence on prion disease incubation time have been mapped in mice (Stephenson *et al.* 2000; Lloyd *et al.* 2001). Although the genes responsible for these mouse quantitative trait loci have not yet been identified, their human orthologues may well play a key role in phenotype determination and may be polymorphic with significant differences within and between Europeans and the Fore.
